# Follow-Up During Early Infancy of Newborns Diagnosed with Subcutaneous Fat Necrosis

**DOI:** 10.4274/jcrpe.355

**Published:** 2011-12-06

**Authors:** Mustafa Ali Akın, Leyla Akın, Dilek Sarıcı, İbrahim Yılmaz, Süleyman Balkanlı, Selim Kurtoğlu

**Affiliations:** 1 Erciyes University, Faculty of Medicine, Department of Pediatrics, Division of Neonatology, Kayseri, Turkey; 2 Erciyes University, Faculty of Medicine, Department of Pediatrics, Division of Pediatric Endocrinology, Kayseri, Turkey; 3 Erciyes University, Faculty of Medicine, Department of Pathology, Kayseri, Turkey; 4 Erciyes University, Faculty of Medicine, Department of Pediatrics, Kayseri, Turkey; 5 Erciyes University, Faculty of Medicine, Department of Pediatrics, Division of Neonatology and Pediatric Endocrinology, Kayseri, Turkey; +90 532 561 79 45+90 352 437 58 25mustafaaliakin@hotmail.comErciyes University, Faculty of Medicine, Department of Pediatrics, Division of Neonatology, Kayseri, Turkey

**Keywords:** Subcutaneous fat necrosis, hypercalcemia, follow-up

## Abstract

Subcutaneous fat necrosis of the newborn (ScFN) is an uncommon  condition caused by generalized and/or local tissue hypoperfusion. The skin lesions of ScFN tend to improve spontaneously. However, ScFN may also lead to complications which cause serious problems. The severity of the etiologic factors contributing to the development of the disease determines the severity of complications. Therefore, these patients should be closely monitored for complications, especially for hypercalcemia which may be life-threatening. The severity and duration of hypercalcemia are associated with the extensity of skin lesions.

We present a newborn who developed ScFN as a result of systemic hypotension. The ScFN resolved after the first few weeks of life, but the patient developed mild hypercalcemia during the 4-month follow-up period. The infant was breast-fed during follow-up, and vitamin D prophylaxis was not initiated. The hypercalcemia resolved within four months without any complications. We would like to draw attention to the need to monitor serum calcium levels in these infants and to refrain from initiating vitamin D prophylaxis in the first months of life.

**Conflict of interest:**None declared.

## INTRODUCTION

Subcutaneous fat necrosis of the newborn (ScFN) is a rare condition and its pathophysiology is unknown. It is usually reported in term newborns, but may occur in preterms as well (1,2,3,4). The skin lesions of ScFN can be described as firm and painful subcutaneous nodules, the appearance of which may vary from an erythematous to a violaceous lesion. Typical lesions are mainly localized on the back, buttocks, shoulders, and the cheeks. In general, they are self-limiting and resolve spontaneously within a few weeks to months after the onset (1,2,3). Hypercalcemia, occurring after healing of skin lesions, is the most dangerous complication of ScFN. It can be life-threatening if not treated adequately (1,2,3).

We present the case of a newborn with uncomplicated hypercalcemia resulting from ScFN and its management during the 4 months of follow-up.

## CASE REPORTS

A 3-day-old female newborn was transferred to our hospital due to suspected neonatal sepsis and presence of a small omphalocele. She was born at term by spontaneous vaginal delivery. We learned that she had undergone transient tachypnea as well as upper gastrointestinal bleeding and hypotension after birth. On admission, physical examination revealed mild tachypnea, an omphalocele, and multiple erythematous skin lesions. Her skin lesions were located on the back, shoulders, the lateral surfaces of the arms, and on the anterior medial side of the right thigh. Laboratory investigations, including complete blood count, serum calcium (Ca), phosphorus (P), alkaline phosphatase (ALP), blood urea nitrogen, creatinine, electrolytes, blood sugar, aspartate transaminase, alanine transaminase, acid-base values, and C-reactive protein levels as well as urinalysis were all normal. Antibiotic therapy was initiated because infection could not be ruled out and terminated after three days  when blood cultures were reported to be sterile. The patient’s tachypnea resolved on oxygen therapy within 24 hours and the omphalocele was corrected surgically on the fifth day of admission. On the first week of admission, the skin lesions transformed into red-purple, firm and painful subcutaneous nodules (Figure 1).  These findings were consistent with the early lesions of fat necrosis of the newborn, and a skin biopsy was performed. Pathological examination of the biopsy material obtained from the lesion border revealed a small number of lymphocytes and focal inflammatory areas consisting of histiocytes (Figure 2a). Additionally, there were radial eosinophilic crystals in the fat cells (Figure 2b). Based on these findings, the patient was diagnosed as ScFN. Spontaneous improvement was observed in the lesions during the first  two weeks following the diagnosis. The baby’s parents were informed about the risk of hypercalcemia and its signs and symptoms. The patient was discharged on the 15th day of admission. Vitamin-D prophylaxis was not initiated. The lesions progressively disappeared over the following four weeks without any complication.  Serum Ca and P levels were monitored twice per month after discharge. Serum Ca, P, ALP, and parathyroid hormone (PTH) levels during admission and follow-up were all within normal ranges and are given in Table 1. A slight increase was noted in  serum Ca levels during the follow-up period. The patient was breast-fed throughout the follow-up period. 

**Figure 1:**
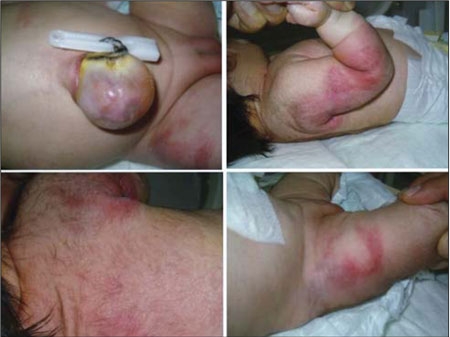
Erythematous skin lesions, red-purple in color were located on the back, shoulders, the external surfaces of the arms, and on the anterior medial side of the thighs

**Figure 2a:**
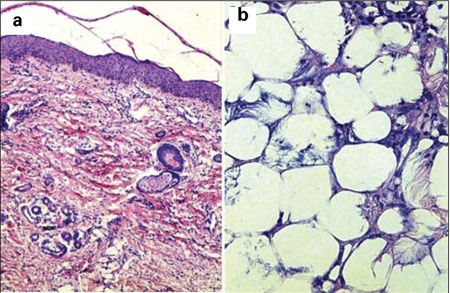
Focal nonspecific inflammation consisting of histiocytes and lymphocytes (a), eosinophilic crystals with radial extension in the necrotic fat cells (b)

**Table 1:**
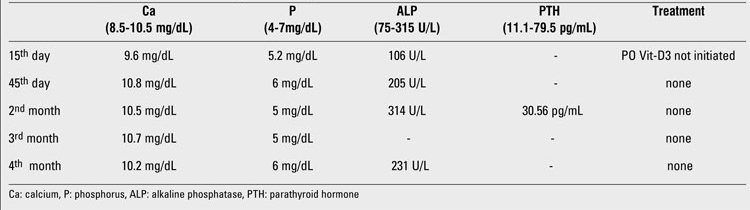
Serum Ca, P, ALP and PTH levels during the follow-up period

## DISCUSSION

ScFN is a rare condition which can be self-limiting in its early period, but which can be life-threatening in its late period. Usually, the history of ScFN patients  reveals a systemic and/or local hypoxia-hypoperfusion episode during the perinatal period. Possible risk factors for ScFN are perinatal asphyxia, meconium aspiration, cord accidents, hypothermia-cold exposure, hypoglycemia and lactic acidosis (1,2,3). ScFN may also develop as a complication of therapeutic hypothermia applied either in newborns with perinatal asphyxia or in newborns undergoing surgical procedures (5,6,7). Maternal conditions which lead to disrupted placental perfusion such as pre-eclampsia, smoking, cocaine exposure, and diabetes may also cause ScFN (1,2,3,7). Our patient had no history of perinatal asphyxia or obstetric trauma and there were no maternal risk factors. However, she had undergone a systemic hypotension episode in the first days of life. 

In ScFN patients, typical skin lesions appear any time after birth up to four weeks after delivery and begin with edema in the affected skin area.  The swelling is followed by hard nodules and plaques (1,2,3). The lesions are very painful (2,8) and, as was also the case in our patient, they resolve spontaneously within a few weeks. In some cases, as the skin lesions heal, they lead to fat tissue atrophy, fibrosis, scarring or ulcers (1,2,3). At admission, our patient had erythematous lesions that transformed into red-purple, firm and painful subcutaneous nodules at the end of the first week (Figure 1). 

Deep hemangioma, lipoedema (cellulite), erysipelas, histiocytosis, fibromatosis, rhabdomyosarcomas and neonatal sclerema must be considered in the differential diagnosis of ScFN. Neonatal sclerema may occur concomitantly with ScFN (1,2,3,7). Definitive diagnosis of ScFN requires histopathological assessment, which will reveal radially arranged fat cells seen as needle-shaped crystals, multinucleated giant cells forming the granulomatous structure, fibroblasts, numerous histiocytes, and small amounts of lymphocyte infiltration (1,2,3,7,9). In our patient, also the diagnosis of ScFN was confirmed by skin biopsy. 

The size and severity of the lesions as well as the complications depend on the etiologic factors. Conditions accompanied by generalized hypoxia-hypoperfusion cause the most serious problems (1,2,3). Other than local skin problems, thrombocytopenia, hypoglycemia, hypertriglyceridemia and hypercalcemia constitute the expected complications of ScFN. All complications, except hypercalcemia, are self-limiting disorders and rapidly respond to treatment. There were no complications except for mild elevation of blood calcium level in our patient. 

Hypercalcemia is the most frequently reported and frightening complication  in ScFN, occurring in 28-69% of the  cases (1,2,3). The pathogenesis of the hypercalcemia is attributed to the necrosis that occurs in the damaged immature fat tissue caused by granulomatous cell infiltration. These macrophages in the area of infiltration lead to increased 1,25-dihydroxyvitamin D3 [1,25(OH)2D3] production, which, regardless of PTH level, leads to increased intestinal absorption of calcium (1,2,3,7).  Release of calcium from necrotic fat cells, increased osteoclastic activity due to elevated PTH and effect of local prostaglandins (PgE2) have been reported as other suspected mechanisms leading to hypercalcemia (1,2,3). The severity and duration of hypercalcemia are related to the severity and duration of skin lesions. Hypercalcemia usually develops in the 4th to 6th weeks of life, when the skin lesions begin to resolve and may last up to the 6th month. If the duration of hypercalcemia is prolonged, metastatic calcifications, nephrocalcinosis and renal failure may develop and may lead to life-threatening cardiac problems   (1,2,3,7,9). The close monitoring of serum calcium levels is imperative in ScFN patients and their parents should be informed about the symptoms of hypercalcemia, such as irritability, anorexia, constipation, and failure to thrive (7,8). 

In conclusion, patients with ScFN should be followed closely in the first months of life, since the complications can be life-threatening. Also, these infants should not receive vitamin D for rickets prophylaxis in the first 6 months.

**Figure 1:**
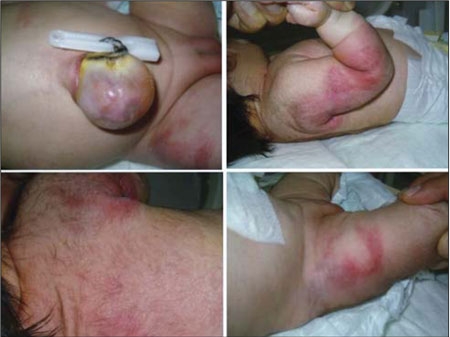
Erythematous skin lesions, red-purple in color were located on the back, shoulders, the external surfaces of the arms, and on the anterior medial side of the thighs
